# An RNA-sequencing transcriptome of the rodent Schwann cell response to peripheral nerve injury

**DOI:** 10.1186/s12974-022-02462-6

**Published:** 2022-04-30

**Authors:** Amanda Brosius Lutz, Tawaun A. Lucas, Glenn A. Carson, Christine Caneda, Lu Zhou, Ben A. Barres, Marion S. Buckwalter, Steven A. Sloan

**Affiliations:** 1 Department of Developmental Biology, Stanford University School of Medicine, Stanford, CA 94305-5125 USA; 2 Department of Neurobiology, Stanford University School of Medicine, Stanford, CA 94305-5125 USA; 3 Department of Neurology and Neurological Sciences, Stanford University School of Medicine, Stanford, CA 94305-5125 USA; 4 Department of Neurosurgery, Stanford University School of Medicine, Stanford, CA 94305-5125 USA; 5 Department of Obstetrics and Gynecology, Inselspital, Bern University Hospital, University of Bern, Bern, Switzerland; 6 Department of Human Genetics, Emory University, 30322 Atlanta, Georgia; 7 Department for BioMedical Research (DBMR), University of Bern, Bern, Switzerland

**Keywords:** Schwann cell, Macrophage, Neuroinflammation, Transcriptome, Repair cell, Injury response, Peripheral nerve, Regeneration

## Abstract

**Background:**

The important contribution of glia to mechanisms of injury and repair of the nervous system is increasingly recognized. In stark contrast to the central nervous system (CNS), the peripheral nervous system (PNS) has a remarkable capacity for regeneration after injury. Schwann cells are recognized as key contributors to PNS regeneration, but the molecular underpinnings of the Schwann cell response to injury and how they interact with the inflammatory response remain incompletely understood.

**Methods:**

We completed bulk RNA-sequencing of Schwann cells purified acutely using immunopanning from the naïve and injured rodent sciatic nerve at 3, 5, and 7 days post-injury. We used qRT-PCR and in situ hybridization to assess cell purity and probe dataset integrity. Finally, we used bioinformatic analysis to probe Schwann cell-specific injury-induced modulation of cellular pathways.

**Results:**

Our data confirm Schwann cell purity and validate RNAseq dataset integrity. Bioinformatic analysis identifies discrete modules of genes that follow distinct patterns of regulation in the 1st days after injury and their corresponding molecular pathways. These findings enable improved differentiation of myeloid and glial components of neuroinflammation after peripheral nerve injury and highlight novel molecular aspects of the Schwann cell injury response such as acute downregulation of the AGE/RAGE pathway and of secreted molecules Sparcl1 and Sema5a.

**Conclusions:**

We provide a helpful resource for further deciphering the Schwann cell injury response and a depth of transcriptional data that can complement the findings of recent single cell sequencing approaches. As more data become available on the response of CNS glia to injury, we anticipate that this dataset will provide a valuable platform for understanding key differences in the PNS and CNS glial responses to injury and for designing approaches to ameliorate CNS regeneration.

**Supplementary Information:**

The online version contains supplementary material available at 10.1186/s12974-022-02462-6.

## Background

**Fig. 1:**
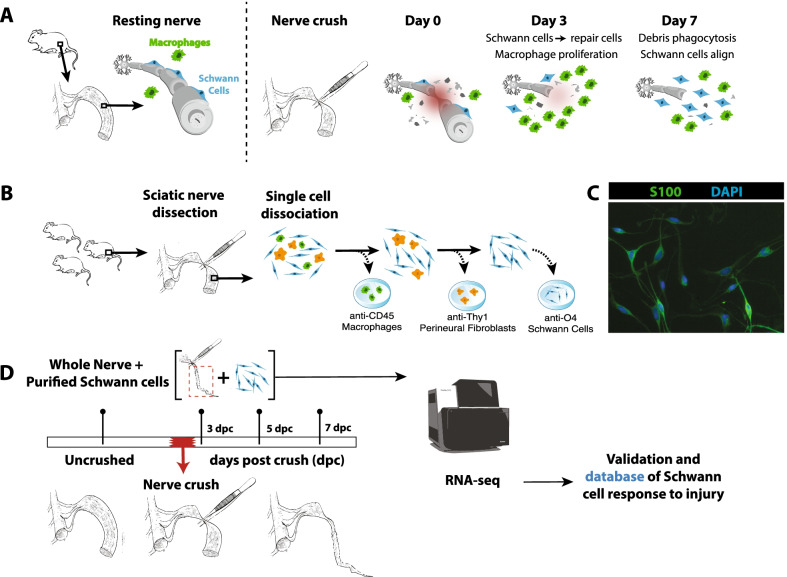
Purification of Schwann cells after sciatic nerve crush. **A** Schematic of local response to sciatic nerve crush. Immediately after crush (day 0), debris and inflammatory cytokines are abundant. By 3 days, macrophages proliferate at the site of injury and Schwann cells adopt a reparative phenotype. 7 days after crush, most debris has been engulfed and Schwann cells align. **B** Purification of Schwann cells by immunopanning. Macrophages and perineural fibroblasts were depleted with anti-CD45 and anti-Thy1 antibodies, respectively. Schwann cells were positively selected by anti-O4 hybridoma. **C** Purified Schwann cells in culture after immunopanning. **D** Experimental timeline. Sciatic nerve crush occurred at day 0. At days 3, 7, and 7 post-crush, both whole nerve and purified Schwann cells were collected for downstream RNA-sequencing

Like the CNS, the peripheral nerve is protected within several layers of connective tissue and behind a diffusion barrier, the blood–nerve barrier. Within the epineurium, the peripheral nerve is composed of endoneurial fibroblasts, endothelial cells, pericytes, resident macrophages, Schwann cells, and efferent and afferent axons [12]. Following breakdown of the blood–nerve barrier the canonical process of Wallerian degeneration ensues, recruiting additional cell types into the nerve. Neutrophils occupy the nerve 1–2 days after injury followed by invasion by hematogenous macrophages, which persist until approximately 2 weeks after injury [12] (Fig. 1A).

Understanding the mechanisms underlying the remarkable ability of the PNS to regenerate after injury has been a topic of intense interest for decades. The Schwann cell, a neural crest-derived glial cell type comprising 80% of the cells in the uninjured nerve has long been recognized as a key player in PNS regeneration, undergoing a remarkable cellular transformation after nerve injury and assuming multiple pro-regenerative functions during recovery of the injured nerve [18]. While the Schwann cell transformation after nerve injury was long thought to represent de-differentiation to a more immature state, we now know that post-injury Schwann cells are distinct from their precursors. Genetically and morphologically, post-injury Schwann cells, or “Repair cells”, are unique, transdifferentiated cells that facilitate tissue rebuilding. Repair cells turn off genes for myelination and activate those for diverse features including upregulation of growth-promoting proteins (GDNF, artemin, BDNF and others), innate immune activation (secretion of cytokines TNFα, LIF, IL1α, IL1β, and MCP-1), structural reorganization, and myelin debris clearance [18, 20, 21]. Numerous recent studies have made considerable progress towards precisely defining the molecular changes behind the Schwann cell injury response, implicating key transcriptional regulators and signaling pathways in this transformation [1, 5, 18, 33].

Until recently, knowledge of global changes in Schwann cell gene expression during the 1st week after injury was largely based on data obtained from whole sciatic nerve before and after crush or transection injury [27, 32, 44, 49]. This data, however, does not allow for distinction between Schwann cell-specific transcriptomic changes and gene expression in other cell types within the nerve, particularly in hematogenous macrophages, which flood the nerve in large numbers beginning 3 days after injury [32]. Purification of Schwann cells away from the other cell types of the peripheral nerve is essential to gain a clear view of the Schwann cell response to injury. Another valuable resource in the field has been microarray data from neonatal Schwann cells in which Erk signaling is activated to mimic injury [33]. While also instructive, this dataset depicts Schwann cells purified at an early developmental timepoint, cultured in vitro, and activated in an in vitro environment in the absence of axon contact, and is thus unlikely to recapitulate all aspects of in vivo transdifferentiation. Circumventing the need for cell purification, several groups have recently performed single cell transcriptomic analysis of the rodent sciatic nerve following autoimmune and mechanical injury [22, 40, 46]. These studies provide exciting insight into heterogeneity of post-injury gene expression changes in Schwann cells and other cell peripheral nerve cell populations, but lack the sequencing depth to identify more subtle responses to injury. Thus, we view this work as a fundamental new resource that will complement the excellent studies above.

Here, we provide a high-resolution RNAseq transcriptome of Schwann cells acutely purified from the uninjured and injured rat sciatic nerve at 3, 5, and 7 days after nerve crush injury at postnatal day (P)18 [29]. In addition, we provide RNAseq analysis of the whole sciatic nerve at the same timepoints. To our knowledge, this is the first deep sequencing RNAseq dataset of purified Schwann cells at across this timespan, a period known to involve dynamic changes in Schwann cell structure and function. Bioinformatics analysis of our dataset highlights dataset integrity and the importance of purifying Schwann cells away from other cell types in the injured nerve to identify Schwann cell-specific gene changes. We identify the most highly up- and down-regulated transcripts at each post-injury timepoint and further classify genes into discrete modules that follow distinct patterns of regulation with respect to time after PNS injury. Interrogation of the genes in these modules identifies aspects of the Schwann cell response to injury with potential for future study. We also compare immune roles of Schwann cells with those of invading macrophages to better differentiate injury responses in these two key cell types. To ensure widespread distribution of our dataset, we have incorporated these data in a user-friendly website previously created by Ben Barres’ lab (www.BrainRNAseq.org) that provides a simple and searchable platform for browsing gene expression data. Our dataset provides a helpful resource for further deciphering the Schwann cell injury response. Furthermore, as additional data become available on the response of CNS glia to injury, we anticipate that this dataset will provide a valuable platform for understanding key differences in the PNS and CNS glial responses to injury and for designing approaches to ameliorate CNS regeneration.

## Methods

### Vertebrate animals

All procedures involving animals were conducted in conformity with Stanford University guidelines and are in compliance with national and state laws and policies according to APLAC protocol 10726.

### Sciatic nerve crush injury

All surgical experiments were performed under 2.5% isoflurane on rats at postnatal day 18. Sciatic nerve crush injury was performed as previously described [30]. Briefly, rats were anesthetized using isoflurane. Upper thigh was shaved and sterilized using isopropanol. A 1-cm incision was made using a scalpel and nerve was visualized via blunt dissection using forceps. The left sciatic nerve was crushed at mid-thigh for 5 s using forceps marked with sterile graphite to mark the crush site. Carprofen (5 mg/kg subcutaneous) was administered for analgesia. No sham operation was performed prior to the collection of naïve nerves.

### Purification of Schwann cells from the naïve and injured sciatic nerve

10–20 sciatic nerves were used for the generation of each sample of purified cells. For injured nerves, sciatic nerve tissue was collected beginning proximally 1 mm distal to the graphite-marked crush site and extending distally along the tibial nerve branch as far as possible approaching the medial calcaneus. The proximal portion of the peroneal nerve branch was included when intact dissection was possible. Schwann cells were isolated from the entirety of the harvested nerve segment. For uninjured nerves, the anatomically equivalent nerve segment was isolated. Schwann cells were acutely purified using immunopanning from the injured or uninjured rat sciatic nerve according to Lutz [29]. Briefly, following enzymatic digestion using collagenase/dispase, nerves were mechanically dissociated to create a single cell suspension. Myelin debris was removed using a nitex filter and BSA cushion. Sequential immunopanning first depleted unwanted cell populations (CD45 and Thy1) and then selected for Schwann cells (O4). Performing depletion of other cell populations prior to positive selection of Schwann cells ensures optimal Schwann cell purity. Yields per nerve ranged from 10 to 20,000 cells for uncrushed nerves to approximately 100,000 cells per nerve after nerve crush injury.

### Immunocytochemistry of immunopanned cells

Cultured cells were fixed with 4% paraformaldehyde for 10 min at room temperature 24 h after immunopanning and then permeabilized and blocked with 10% goat serum, 0.2% TritonX-100. Cells were labeled using antibodies to goat Iba1 (Abcam) and mouse S100 (Sigma s2532) as well as Cellmask (ThermoFisher H34558) and DAPI.

### RNA isolation from whole nerves

RNA from whole nerves was purified according to the RNeasy Fibrous Tissue kit. For injured nerves, only the nerve segment distal to the site of crush injury was included. For uninjured nerves the anatomically equivalent nerve segment was isolated. A minimum of 10 nerves was used for the generation of each RNA sample. RNA quality was verified through Bionalyzer analysis. All samples submitted for RNASeq had a RIN of 8 or above.

### RNA isolation from purified Schwann cells

RNA from purified Schwann cells was isolated at the end of the immunopanning procedure by scraping the O4-positive panning dish according to the instructions of the RNeasy MICRO kit. RNA quality was verified through Bionalyzer analysis. All samples submitted for RNASeq had a RIN of 8 or above.

### RNA-seq library construction and sequencing

Total RNA that passed QC standards (RIN > 8.0 as assessed by bioanalyzer) proceeded to library synthesis using the Ovation^®^ RNA-seq system V2 (Nugen 7102). First and second-strand cDNA synthesis and SPIA amplification were performed following the manufacturer’s instructions, and cDNA was fragmented with a sonicator (Covaris S2) using the following parameters: duty cycle 10%, intensity 5, cycles/burst 100, time 5 min. We then used the Next Ultra RNA-seq library prep kit for Illumina (NEB E7530) and NEBNext^®^ multiplex oligos for Illumina^®^ (NEB E7335 E7500) to perform end repair, adaptor ligation, and 5–6 cycles of PCR enrichment according to manufacturer’s instructions. The quality of the libraries were then assessed by bioanalyzer and qPCR and high-quality libraries were sequenced by the Illumina NextSeq sequencer to obtain 150-bp pair-end reads to a minimum depth of 20 million reads per sample. All libraries passed standard QC metrics (average *Q*-scores > 35, mapping % > 80).

### Bioinformatic processing of RNAseq data

FASTQ files were first groomed using FASTQ groomer and then mapped using TopHat2, which invokes Bowtie as an internal read mapper. The paired end option was selected and rat genome Rnor_6.0 was used as the reference genome. We then ran Cufflinks to assemble transcripts and estimate expression level as fragments per kilobase of transcript sequence per million mapped fragments (FPKM). All subsequent data analysis was performed using R software (https://www.r-project.org/). We deposited all raw .fastq files from the RNA-seq data in the National Center for Biotechnology Information (NCBI) Gene Expression Omnibus (GEO, Accession number GSE177037). Unsupervised hierarchical clustering of FPKM data was performed in R using the top 2000 most variable genes with the following settings: Linkage; average, ColumnPdistance; Spearman, standardized across rows.

### Differential expression and pathway analysis

To assess the functional difference of genes post-nerve crush, we carried out pathway analysis on the top 2000 differentially expressed genes at each timepoint. FASTQ files were aligned using STAR [8] (v2.7.1) to the Rnor 6.0 reference genome and counts were generated using RSEM [26] (v1.3.1). Differentially expressed genes were determined using the DEseq2 [28] (v1.26) package within the R environment. Differences were visualized as volcano plots using the EnhancedVolcano package [4] (v1.80) from Bioconductor. Pathway analysis was carried out using Bioconductor package pathfindR [41] with *p* < 0.01 for both the Gene Ontology-Molecular Function and KEGG databases.

### Weighted gene co-expression network analysis (WGCNA)

WGCNA analysis was performed using the R package ‘‘WGCNA’’ [24, 25]. The power value within the blockwise consensus module was determined by examining the soft-threshold-mean-connectivity curve (*p* = 21). The minimum WGCNA module size was 30 genes. Modules with < 0.25 similarity were merged.

### qRT-PCR validation

We designed primers spanning exon–exon junctions to avoid amplification of genomic DNA. The primers used are listed in Additional file 3. We performed 40 cycles of amplification for all samples. The specificity and efficiency of all primers were first validated using gel electrophoresis and qPCR. The determination of each gene’s CT in qPCR was performed in triplicate. When determining fold changes in gene expression across post-injury timepoints, the CT of each gene was normalized according to the CT of the housekeeping gene in the same sample.

### In situ hybridization validation

Full-length rat cDNA expression plasmids for the genes analyzed were linearized and transcribed with T7, Sp6, or T3 RNA polymerases to generate fluorescein (FITC)-labeled single-stranded anti-sense riboprobes according to manufacturer’s instructions (11175025910; Roche) and purified over G-50 columns (GE Healthcare) (Additional file 4). Fresh frozen 12-µm-thick nerve cryosections from P18 rats were processed as described [50] for in situ hybridization and immunohistochemistry using a mouse S100 antibody (Sigma s2532).

## Results

### Schwann cell RNA purification and RNAseq transcriptional profiling

To gain insight into the Schwann cell response to peripheral nerve injury, we generated an RNAseq resource for Schwann cells acutely purified from the naïve and injured rat sciatic nerve at 3, 5, and 7 days post-crush. For comparison, we also generated RNAseq data for the naïve rat sciatic nerve (Fig. 1B, D) (Additional file 1). We chose to examine gene changes over the first 7 days after mouse nerve crush injury because this time period encompasses the key events of Wallerian degeneration including axon degeneration, myelin debris formation, Schwann cell transdifferentiation, Schwann cell proliferation, the major phase of Schwann cell-mediated debris clearance, macrophage recruitment, and the onset of macrophage-mediated debris clearance [12]. We aimed to capture the transcriptional changes associated with these events. The full dataset of purified Schwann cells and whole nerve segments can be found in Additional file 4.

To assess the purity of our O4-immunopanned Schwann cells we used immunocytochemistry and expression of cell type-specific genes. Immunocytochemistry of cells purified from uncrushed nerves using the Schwann cell-specific marker S100 demonstrated a > 95% pure population of Schwann cells with typical spindle-like morphology 24 h after immunopanning (Fig. 1C). Both O4 and S100 are broadly expressed in the Schwann cell lineage [19].

**Fig. 2:**
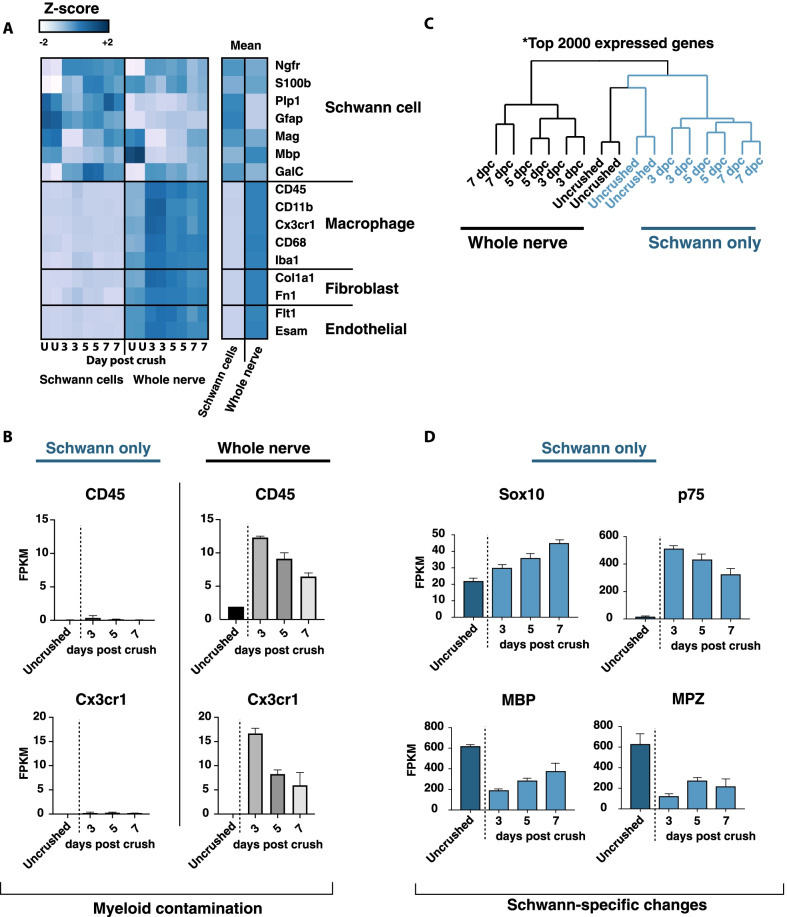
Purity of Schwann cell isolation. **A** Expression of typical Schwann cell, macrophage, fibroblast, and endothelial markers in whole nerve samples and purified Schwann cells at each day post-crush. **B** Expression of macrophage markers, CD45 and Cx3cr1, in purified Schwann cells as compared to whole nerve. Purified Schwann cells are almost completely devoid of myeloid markers. **C** Hierarchical clustering of all samples (top 2000 most variable genes; Linkage, average; ColumnPdistance, Spearman). **D** Schwann cell response to injury confirmed by expected upregulation of Sox10 and p75, and downregulation of myelin genes MBP and MPZ

Examination of FPKM values for known cell type-specific genes in our whole nerve and purified Schwann cell RNAseq data revealed high enrichment for Schwann cell-specific transcripts and depletion of transcripts from potential contaminating cell types. Specifically, we demonstrated the depletion of S100 expressing perineurial glia and satellite glial transcripts in our purified Schwann cell samples (Additional file 9) [13, 39] In addition, we were able to deplete macrophage, endothelial cell, and fibroblast cell type-specific markers at all timepoints examined (Fig. 2A). Given the large influx of myeloid cells into the nerve after injury, we wanted to more closely examine the possibility that our Schwann cell samples post-injury might be contaminated by infiltrating hematogenous myeloid cells. Based on absolute FPKM, we confirmed that while myeloid markers indeed increase significantly in the whole nerve transcriptome post-injury, these were near completely depleted in our purified Schwann cell samples (Fig. 2B).

Supporting the notion that purification of Schwann cells away from the sciatic nerve is crucial for the analysis of the Schwann cell injury response in order to avoid confounding transcriptome changes in other cell types after nerve injury, unsupervised hierarchical clustering demonstrated that expression profiles of naïve whole nerve and naïve Schwann cells cluster together while profiles of injured whole nerve and injured Schwann cells form distinct clusters (Fig. 2C). As a further quality control measure, we examined genes known to be up- or down-regulated in Schwann cells in the injured nerve. As expected, our RNAseq profiles revealed persistent expression of lineage marker Sox10, a marked downregulation of myelin genes MBP and MPZ and upregulation of immature and non-myelinating Schwann cell marker, NGFR after nerve crush injury (Fig. 2D).

### Validation of RNAseq results

**Fig. 3:**
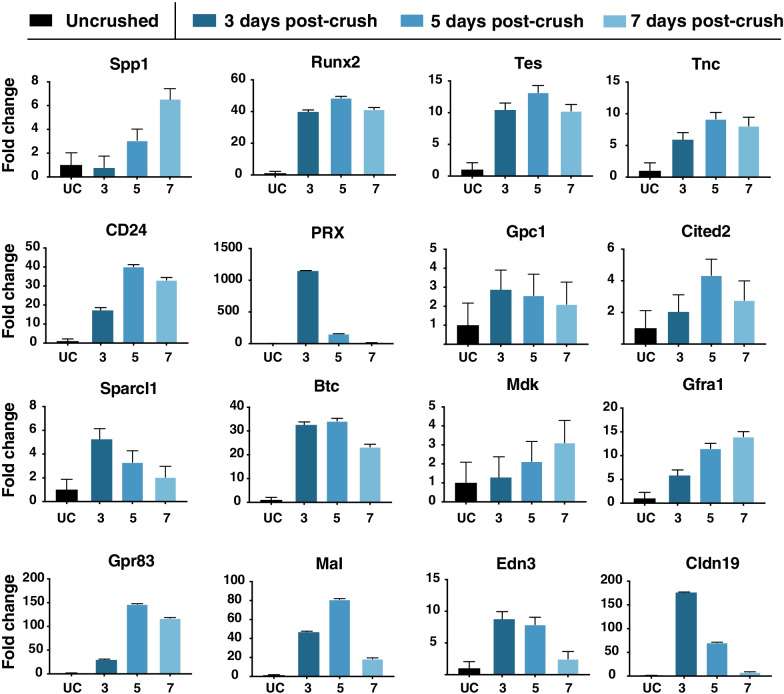
qRT-PCR validation. **A** Differentially expressed genes from the RNA-seq analysis were selected for validation by qRT-PCR. Schwann cells were purified from uncrushed or post-injury sciatic nerves and cDNA was generated for primer-based amplification using exon-spanning primers. UC: uncrushed. Spp1: secreted phosphoprotein 1, Runx2: runx family transcription factor 2, Tes: Testin LIM domain protein, Tnc: Tenascin C, PRX: periaxin, Gpc1: Glypican 1, Cited2: Cbp/P300 interacting transactivator with Glu/Asp rich carboxy-terminal domain 2, Sparcl1: SPARC Like 1, Btc, betacellulin, Mdk: midkine, Gfra1: GDNF family receptor alpha 1, Gpr83: G protein-coupled receptor 83, MaI: Mal, T cell differentiation protein, Edn3: endothelin 3, Cldn19: claudin 19

**Fig. 4:**
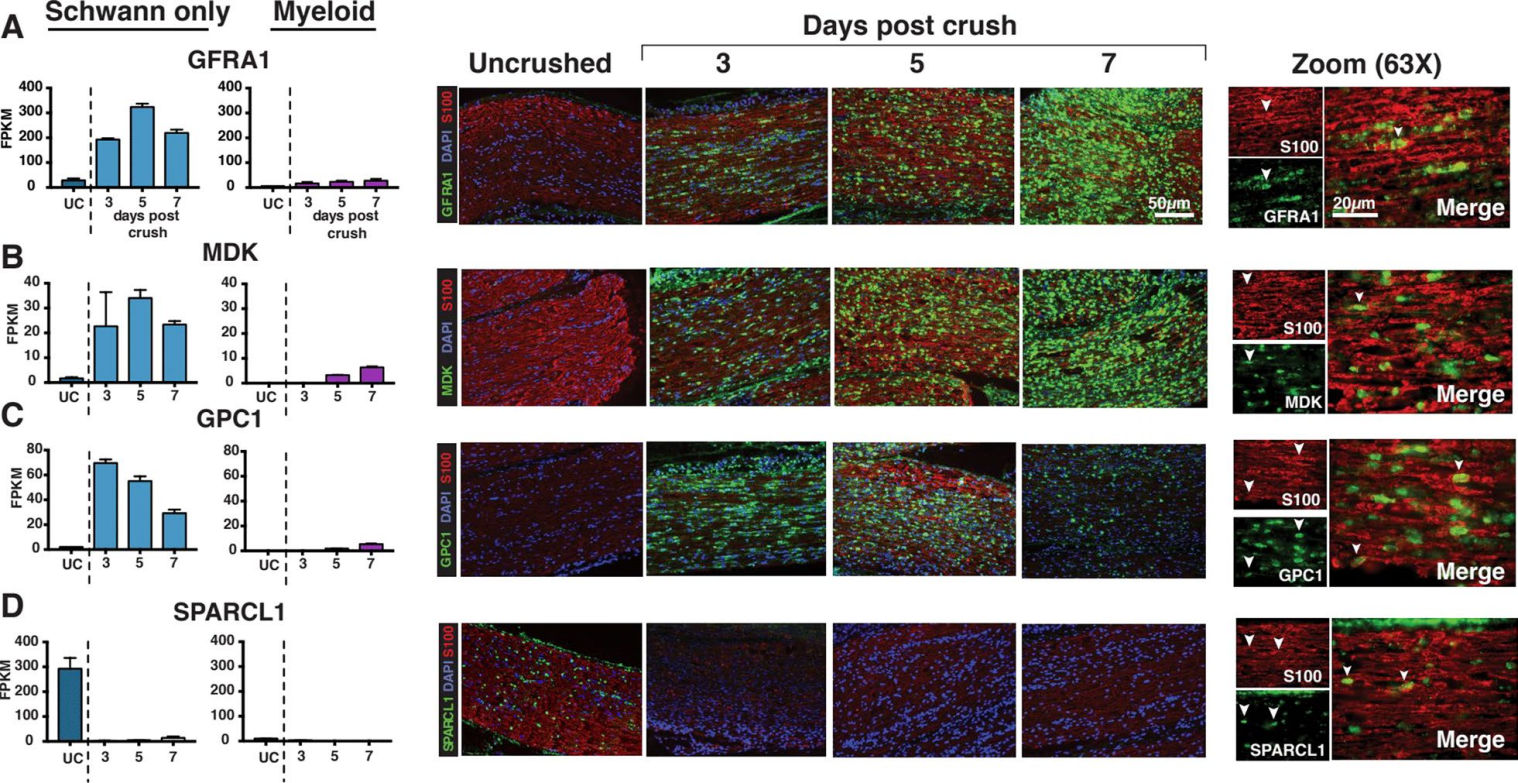
In situ hybridization spatial validation. **A–D** Four genes (GFRA1, MDK, GPC1, and SPARCL1) were selected for validation based on their clear response to injury in Schwann cells and lack of change in myeloid populations after injury. Sciatic nerves were counterstained with S100 (red) to confirm Schwann cell localization of expression changes. All four genes exhibit expected temporal expression patterns within the nerve (GFRA1, MDK, GPC1 increasing and SPARCL1 decreasing). Right, 63× zoom. Arrowheads highlight examples of S100 colocalization with the in situ probe for each candidate gene. Scales = 50 μm (left images), and 20 μm (right images)

Next, we examined the validity of our Schwann cell RNAseq transcriptome datasets by two orthologous methods. We began by identifying a list of transcripts that appeared from our RNAseq data to be highly regulated after nerve injury. To be selected, transcripts had to attain an FPKM of at least 30 FKPM at any timepoint, and were either highly upregulated (sevenfold or more increase in FPKM) or down-regulated (sevenfold or more decrease in FPKM) during the course of injury. First, we used qRT-PCR to validate a subset of these top differentially expressed Schwann cell genes in the week following nerve crush injury. We found consistent agreement between the qRT-PCR data and our RNAseq findings (Fig. 3). Following qRT-PCR validation, we used in situ hybridization to confirm Schwann cell origin for the transcriptional changes observed. We designed fluorescence in situ hybridization probes directed against a subset of the top up- and down-regulated transcripts (Additional file 2), selected based on (a) biological interest and (b) amenability to specific probe design. We then performed simultaneous immunohistochemistry with an antibody directed against the Schwann cell marker, S100 (Fig. 4A–D) to validate Schwann cell-specificity for each in situ probe.

Our in situ hybridization confirmed the anticipated changes in gene expression in S100-positive Schwann cells, with putative upregulated transcripts exhibiting no signal co-localization with S100 immunostaining in uninjured nerves and co-localization with S100-positive Schwann cells post-injury. Similarly, putative down-regulated transcripts (SPARCL1) exhibited signal co-localized with S100 immunostaining prior to injury and were no longer detectable in immunolabeled Schwann cells after injury (Fig. 4).

For a subset of up- and down-regulated transcripts, we observed parallel trends in gene expression in S100-negative cells, in particular macrophages. Therefore, we wanted to further confirm that the transcriptomic changes we denoted originated in Schwann cells, and also differentiate immune responses due to Schwann cell expression of injury-related genes from those mediated by CD45-expressing myeloid cells. We sequenced CD45-positive myeloid cells that were depleted from our sciatic nerve cell suspension during CD45-based immunopanning post-injury. This data revealed that although CD45-positive macrophages exhibit occasional parallel trends in gene expression compared with purified Schwann cells, the level of the expression changes for these genes in this infiltrating population (absolute FPKM) was typically at least an order of magnitude less than in purified Schwann cells (Fig. 4A–D). This finding indicates that Schwann cells relative to macrophages are the primary drivers of gene expression changes for these differentially expressed genes post-injury.

### Analysis of Schwann cell-specific gene expression before and after peripheral nerve injury

**Fig. 5:**
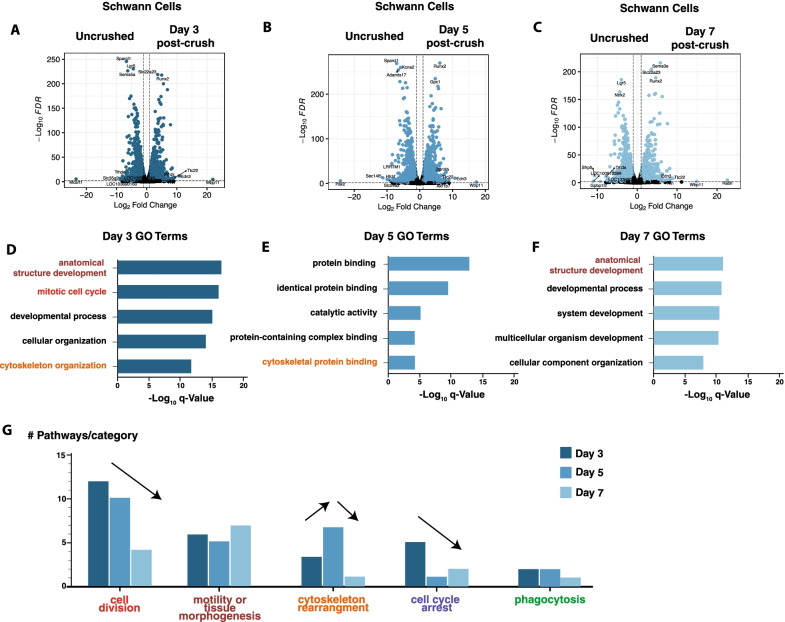
Schwann cell gene changes post-crush. **A–C** Volcano plots of purified Schwann cells at day 3, 5, or 7 post-crush as compared to Schwann cells purified from uncrushed nerves. **D–F** Enriched pathways in Schwann cells from each timepoint based on Gene Ontogeny (GO) and Kyoto Encyclopedia of Genes and Genomes (KEGG) Pathway analysis of the top 2000 most upregulated genes at each post-crush timepoint (all genes with *p*adj < 0.05). **G** Most enriched pathways (or GO categories) at day 3, 5 and 7. GO terms were binned across each time point, with the top five most frequently observed terms presented here. Direction of arrows highlight that some pathways, e.g., cell division and cell cycle arrest, decrease with time post-crush, while others (cytoskeletal rearrangement) peak at intermediate timepoints post-injury

We next used volcano plots to visualize global changes in Schwann cell gene expression at each post-crush timepoint relative to the naïve Schwann cell transcriptome (Fig. 5A–C) (calculated using the DESeq2 package). These comparisons revealed widespread transcriptomic changes (average = 5398, *p*adj < 0.05) in Schwann cells at each time post-crush stage. The number of significant differentially expressed genes (DEGs) was relatively consistent at each timepoint (5495, 5744, and 4955 genes at days 3, 5, 7 after injury), although the composition of these genes differed throughout each stage. The full list of DEGs at each timepoint can be found in Additional file 5.

To identify the most relevant biological pathways and processes induced by nerve crush in Schwann cells, we performed Gene Ontogeny (GO) and Kyoto Encyclopedia of Genes and Genomes (KEGG) Pathway analysis of the top 2000 most upregulated and downregulated genes at each post-crush timepoint (Fig. 5D–F, all genes with *p*adj < 0.05) (Additional files 6 and 7). Among upregulated genes, to determine which biological categories exhibit stage-dependent changes, we quantified how many GO hits were present at each post-injury timepoints (Fig. 5G). While some categories were quite consistent throughout all timepoints (motility, phagocytosis), others displayed significant enrichment at either day 3 (cell division) or day 5 (cytoskeletal rearrangement). The most highly downregulated GO terms at days 3, 5, and 7 after injury consistently relate to extracellular matrix production and cell adhesion (Additional file 7). Our GO analysis of downregulated transcripts also highlighted a strong silencing of ion channel expression, likely reflecting the dismantling of highly specialized Schwann cell membrane regions in close proximity to axons [3, 7, 45] (Additional file 7).

### Using WGCNA to identify co-regulated networks in the Schwann cell injury response

**Fig. 6:**
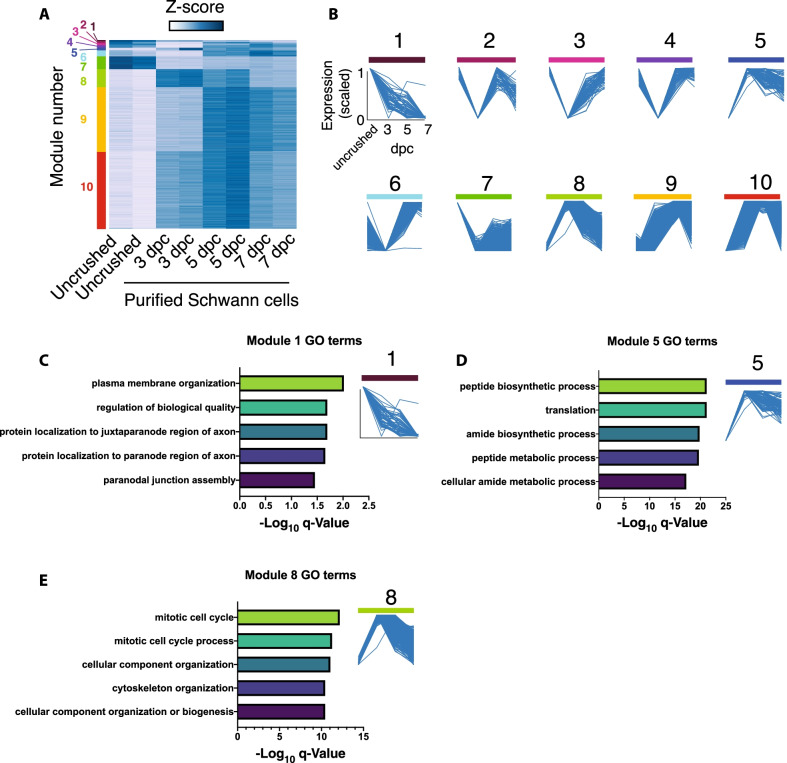
WGCNA of purified Schwann cells after sciatic nerve crush. **A**, **B** WGCNA on purified Schwann cells. The power value within the blockwise consensus module was determined by examining the soft-threshold-mean-connectivity curve (*p* = 21). The minimum WGCNA module size was 30 genes. Modules with < 0.25 similarity were merged to produce 10 final modules. **C**–**E** Enriched GO terms for 3 modules (1, 5, and 8) with distinct expression patterns

Finally, we wondered whether we could identify families of genes who displayed similar expression patterns across all post-injury timepoints within Schwann cells. While this analysis could include consistent up- and down-regulated trends throughout the duration of the injury, it could alternatively involve, for example, groups of genes that peak at 3 days post-injury and then decay, or those that remain low until before increasing at 7 days post-injury. Categorizing genes by these patterns allows for a more nuanced understanding of which pathways share temporal characteristics during injury. Therefore, we performed unsupervised grouping of Schwann cell-specific genes into ten distinct modules using weighted gene co-expression network analysis (WCGNA), each defined by a distinct pattern of gene expression over the examined timecourse (Fig. 6A, B). These clusters can be mined individually to discover potentially novel pathways and groups of Schwann cell genes regulated in distinct patterns after nerve injury. A full list of genes belonging to each module can be found in Additional file 8. To demonstrate the feasibility of clustering injury response genes in these groups, we sampled three modules (1, 5, and 8) that exhibit distinct temporal patterns of expression and asked which pathways were most abundant in each (Fig. 6C–E). Module 1 genes, which decline rapidly after injury, were enriched in genes associated with membrane organization and formation of membrane sub-domains, consistent with dismantling of myelin nodes and paranodes. This is in contrast to module 5 and 8 genes, which spike rapidly after injury and are most associated with metabolic and cell cycle processes, likely reflecting Schwann cell proliferation after injury.

### Acute regulation of PI3K/Akt/mTOR and AGE/RAGE activation in the Schwann cell injury response

Our WCGNA analysis also highlighted the temporal regulation of specific molecular pathways that are of interest for further study. The PI3K/Akt pathway, implicated in multiple studies involving the Schwann cell response to injury, Schwann cell proliferation, and in the initiation of Schwann cell myelination, was grouped into module 8, a cluster of genes for which expression increases sharply by day 3 after injury and then decreases in expression intensity by day 5 and further by day 7 after injury [10, 35, 47]. WGCNA analysis also identified the AGE–RAGE pathway within module 7, a cluster of genes sharply downregulated at 3 days post-injury that gradually increase back towards baseline expression at days 5 and 7 after injury.

## Discussion

The transformation of the Schwann cell in response to peripheral nerve injury has long been recognized as a key feature of the peripheral nerve’s remarkable ability to regenerate and yet the molecular underpinnings of this transformation have remained incompletely understood. Here, we provide an RNAseq dataset on Schwann cells acutely purified from rodent peripheral nerves at multiple timepoints during the 1st week after nerve crush to provide insight into the transcriptional changes underlying the Schwann cell response to injury.

We used published immunopanning methods to purify Schwann cells away from other cell types in the nerve, including resident macrophages, hematogenous immune cells, and endoneurial fibroblasts at postnatal day 18. Several methodological points deserve brief discussion. First, O4 is highly expressed by all Schwann cells (both the myelinating and non-myelinating (Remak)) at rat postnatal day 18 [20]. While it is plausible that some Schwann cells may not bind to our O4 coated panning for technical reasons such as incomplete tissue digestion, we have no reason to believe that we have excluded a biologically meaningful Schwann cell sub-population. Secondly, purification of viable Schwann cells from uninjured whole sciatic nerves is increasingly difficult as the nerve ages. Connective tissue becomes increasingly difficult to digest and myelinating cell processes are increasingly difficult to dissociate from axons without causing Schwann cell death. We chose postnatal day 18 as our initial timepoint because it represents a temporal stage at which both myelinating and unmyelinating Schwann cells are reaching maturity and yet can still be dissociated from the nerve in sufficient numbers [19, 29]. Nonetheless, potential subsequent maturational changes should be taken into account when interpreting our data as representative of the adult nerve. Finally, given that immunopanning is performed at room temperature, we do not anticipate significant changes in cellular gene profiles during the cell purification process, with the exception of immediate early genes. We have confirmed this by comparing transcriptomes of whole nerve with dissociated nerve (without immunopanning).

While recent single cell expression analyses contribute unprecedented granularity to our knowledge of Schwann cell gene expression, they lack the sequencing depth provided by bulk RNAseq to more accurately define and quantify the molecular changes occurring in Schwann cells after injury (~ 1000 genes per Schwann cell detected in Wolbert et al. vs ~ 8000 in this study). Thus, both approaches are valuable tools for understanding the Schwann cell response to injury and should be viewed as complementary to one another. Our initial analysis of our bulk RNAseq results underlines several familiar themes of Schwann cell transdifferentiation, as well as potential avenues for future study. These are outlined below.

### Tissue morphogenesis

There has long been consensus that Schwann cells distal to a site of peripheral nerve injury reorganize to form cellular columns known as Bungner’s bands. The importance of these cellular columns for successful nerve regeneration is also well-established, yet the mechanisms underlying the formation of these bands remains incompletely understood. Elegant studies using lineage tracing analysis have recently provided insight into the Schwann cell sub-type contributions and changes in Schwann cell structure that enable the formation of these columns [2]. The molecular underpinnings of these changes, however, remain unclear. Our GO and KEGG analysis of highly upregulated transcripts revealed a large proportion of terms related to cytoskeletal rearrangement, tissue morphogenesis, and ECM receptor expression. Further mining of these transcriptional changes within our database could yield important insight into the regulation of molecular pathways that direct this aspect of the Schwann cell injury response.

### Phagocytosis

Recent work has highlighted that in parallel with macrophages, Schwann cells contribute to debris clearance within the injured peripheral nerve [5, 15]. Accordingly, our KEGG analysis of genes highly upregulated after injury highlights endocytosis and lysosomal degradation as key upregulated cellular processes in the 1st days after injury. While macrophages express a broad repertoire of phagocytic receptors in the injured nerve, Schwann cell-mediated phagocytosis involves specific upregulation of TAM (Tyro, Axl, Mer) receptors Axl and Mertk [5]. The involvement of lectin-mediated phagocytosis, specifically galectin-3, in Schwann cells debris clearance has also been suggested [37]. The role of this pathway in peripheral nerve repair has however been debated, with galectin-3 knock-out mice exhibiting an unexpected enhanced capacity for peripheral nerve regeneration [34]. Galactose metabolism was one of the most upregulated pathways revealed through our KEGG analysis, suggesting that this the involvement of this pathway in the Schwann cell response to injury may merit further study.

### Extracellular matrix changes

Our transcriptional profiling of the post-injury sciatic nerve also highlights extracellular matrix changes. On the side of infiltrating myeloid cells, we observed a strong upregulation of glycan breakdown enzymes after injury. Two Schwann cell transcripts, Sparcl1 and Sema5a, are among the most significantly downregulated genes within the first week after injury. Sparcl1, or Hevin, a secreted non-neuronal protein is implicated in synapse initiation and remodeling during development and after injury [11]. Most work to date on Sparcl1 has been in the CNS [6, 11, 23]. The magnitude of transcriptional regulation revealed in our RNAseq dataset suggests that this protein is likely playing a role in the Schwann cell injury response beyond synapse regulation at the neuromuscular junction. Sema5a, a thrombospondin repeat-containing axon guidance molecule is known to contribute to CNS axon outgrowth inhibition after injury [14]. The effect of Schwann cell downregulation of Sema5a on PNS axon regeneration has not yet been studied.

### Regulation of PI3K/Akt/mTOR and AGE/RAGE activation

A complex interplay between the Mek/ERK1/2-MAPK and PI3K/Akt/mTOR pathways is suggested in Schwann cell myelination and myelin pathologies [17]. A dual role for the PI3K/Akt/mTOR pathway in Schwann cell development and myelination has been reported, with activation of the pathway required for the inhibition of premature Schwann cell differentiation during development, and then subsequently for positive promotion of myelin growth [10]. Whether this pathway is differentially implicated in developmental myelination versus post-injury re-myelination has not been extensively studied. Our data suggest that activation of PI3K/Akt/mTOR following nerve injury is dramatic at the acute stage, but rapidly declines towards baseline levels by 1 week post-injury. Further study of the temporal regulation of this pathway could suggest an anticipated second wave of expression. Additionally, differential spatial regulation of the PI3K/Akt/mTor signaling between proximal and more distal portions of the regenerating nerve may elucidate differences between developmental and post-injury Schwann cell differentiation and myelination. In our CD45-positive myeloid cell sequencing results, we observed a significant upregulation of MAPK signaling, as reported following CNS injury [48].

The AGE/RAGE pathway has to date been most heavily studied in age-related vascular dysfunction where it has been found to mediate age-related vascular stiffening. The accumulation of AGEs is known to be accelerated in diabetes [9, 38]. Although the AGE receptor RAGE is expressed in Schwann cells, its role in the peripheral nerve has not yet been studied [42]. Recent work shows that age-related changes in Schwann cells contribute to failure of the aged PNS to regenerate to the same extent as the young PNS [36]. Our observation that a decrease in RAGE occurs in parallel with the Schwann cell reprogramming indicates that a silencing of this pathway is at least involved in, if not required for a successful Schwann cell response to injury. Such silencing may not be feasible in the aged and diabetic nerve, where AGEs have heavily accumulated. Failed downregulation of AGE–RAGE signaling could help to explain a defective Schwann cell response to injury in aged nerves and possibly a defective homeostatic nerve repair mechanism in diabetic neuropathy.

### Relevance to human peripheral nerve injury

The ultimate value of findings in rodent models of nerve injury depends on their relevance to human disease. Data on the human Schwann cell response to injury are extremely scarce. Recent studies using degenerating nerve fascicles in vitro, however, demonstrate important parallels between rodent and human Schwann cells [31, 43]. Importantly, human Schwann cells appear to recapitulate key features of murine repair Schwann cells. Namely, human Schwann cells demonstrate adaptive cellular reprogramming after injury including cJUN upregulation and expression of immature Schwann cell markers L1CAM, NEUM and NGFR, downregulation of myelin-associated genes such as MBP and MYP0, and participation in debris clearance [43]. Schwann cells in both species demonstrate a reduction in the Schwann cell injury response with increased age [5, 31]. Interestingly, work on the relative timing of early events in the human Schwann cell injury response indicates that the Schwann cell injury response in humans is delayed in comparison with mice [31]. Specifically, human Schwann cells exhibit delayed myelin degradation, delayed transdifferentiation (slower/less robust silencing of differentiation genes and slower/less robust induction of repair genes) and reduced adaptation of lipid metabolism. The regenerative capacity of the rodent peripheral nerve (4.5 mm/day) is also known to exceed that of the human nerve (1–1.5 mm/day) and could be tightly linked to differences in the Schwann cell injury response [31]. The underpinnings of these differences are not yet well understood. This recent work supports the relevance of work performed in murine models of peripheral nerve injury to human peripheral nerve repair. Additional studies in human Schwann cells are needed to deepen our understanding of the similarities and differences between Schwann cell injury responses in these two species.

## Future perspectives

A fascinating aspect of Schwann cell biology is the bifurcation of the Schwann cell lineage during development to form two distinct populations of mature Schwann cells, non-myelinating (Remak) cells and myelinating Schwann cells. Our understanding of the gene expression differences between Remak and myelinating Schwann cells has until very recently remained rudimentary, based on small handful of cell-subtype specific markers such as GFAP and myelin proteins [19]. Using single cell sequencing and genetic labeling, Gerber and colleagues have very recently provided the first transcriptome data for the developing Schwann cell lineage that includes separation of the Remak and myelinating Schwann cell populations [13]. For years, despite advances in other aspects of the Schwann cell response to injury, how the roles of these two Schwann cell sub-types compare during peripheral nerve regeneration has remained elusive. Recent studies have provided the first insights into the morphological contributions of myelinating and non-myelinating Schwann cells after nerve transection, demonstrating that both myelinating and non-myelinating Schwann cells have the capacity to change their shape and transform into elongated repair cells and that repair cells in turn can shorten dramatically following axon regeneration and form myelin internodes [16]. Applying single cell sequencing technologies akin to the approach of Suter et al. to the injured nerve’s myelinating and Remak populations will build upon available transcriptomes, and notably on the data we present here to further elucidate the transcriptional basis of Schwann cell transdifferentiation, heterogeneity of repair cells, maintenance of the repair cell state, and Schwann cell re-differentiation.

## Conclusions

The transcriptomic data presented here provide a valuable resource to peripheral nerve biologists to fuel novel hypotheses and uncover new pathways underlying the Schwann cell injury response. In addition to the molecules highlighted above, we anticipate the discovery of to date un-appreciated pathways and proteins using our dataset that will enable mechanistic advances in our understanding of how these remarkable cells transform after nerve injury. Confirmation of our transcription-level changes at the protein level followed by knock-out or overexpression experiments has the potential to yield answers to outstanding questions in the field of Schwann cell biology. For example, to further our understanding of why the PNS regenerates so well, and to generate new molecular handles for both understanding CNS regeneration failure and designing strategies to overcome it.

## Supplementary Information

**Additional file 1.** PCA of all RNA-seq samples. (A) PCA of all samples colored by day post-crush. Circles indicate whole nerve samples and triangles represent Schwann cell purified samples. (B) PCA of Schwann cell samples alone, colored by time point.

**Additional file 2. **Generation of in situ hybridization probes. RNA probes used for in situ hybridization validation. Expected probe lengths are listed beneath each column.

**Additional file 3. **qRT-PCR primer sequences (rat) used to validate RNAseq findings.

**Additional file 4.** All FPKM data for uncrushed (day 0) and crushed (days 3, 5, 7 post-crush) samples. This includes whole nerve and purified Schwann cells. Also included is dissociated sciatic nerve to help identify genes that change from the dissociation process, itself.

**Additional file 5.** All differentially expressed genes and their *p*-values (and *p*-adjusted) in Schwann cells at days 3, 5, and 7 post-crush, as compared to day 0 (uncrushed).

**Additional file 6. **Table of enriched gene pathways in purified Schwann cells at days 3, 5, and 7. First two sheets show pathways across all timepoints and the subsequent sheets highlight statistics of each pathway separated by timepoints.

**Additional file 7. **Table of downregulated gene pathways in purified Schwann cells at days 3, 5, and 7.

**Additional file 8. **Module assignments (from the WGCNA) for each gene.

**Additional file 9. **Analysis of markers of different S100-expressing cell populations.

## Data Availability

To ensure widespread distribution of our RNAseq dataset, we have incorporated these data in a user-friendly website previously created by Ben Barres’ lab (www.BrainRNAseq.org) that provides a simple and searchable platform for browsing gene expression data.

## Abbreviations

CNS: Central nervous system
FPKM: Fragments per kilobase of transcript sequence per million mapped fragments
GEO: Gene Expression Omnibus
GO: Gene Ontogeny
KEGG: Kyoto Encyclopedia of Genes and Genomes
NCBI: National Center for Biotechnology Information
P: Postnatal day
PNS: Peripheral nervous system

## References

[1] Arthur-Farraj PJ, Latouche M, Wilton DK, Quintes S, Chabrol E, Banerjee A (2012). c-Jun reprograms Schwann cells of injured nerves to generate a repair cell essential for regeneration. Neuron.

[2] Arthur-Farraj PJ, Morgan CC, Adamowicz M, Gomez-Sanchez JA, Fazal SV, Beucher A (2017). Changes in the coding and non-coding transcriptome and DNA methylome that define the schwann cell repair phenotype after nerve injury. Cell Rep.

[3] Baker MD (2002). Electrophysiology of mammalian Schwann cells. Prog Biophys Mol Biol.

[4] Blighe K, Rana S, Lewis M. EnhancedVolcano: publication-ready volcano plots with enhanced colouring and labeling. R package version 1.10.0. 2021.

[5] Brosius Lutz A, Chung WS, Sloan SA, Carson GA, Zhou L, Lovelett E (2017). Schwann cells use TAM receptor-mediated phagocytosis in addition to autophagy to clear myelin in a mouse model of nerve injury. Proc Natl Acad Sci USA.

[6] Chen S, Zou Q, Chen Y, Kuang X, Wu W, Guo M (2020). Regulation of SPARC family proteins in disorders of the central nervous system. Brain Res Bull.

[7] De Logu F, Nassini R, Materazzi S, Carvalho Gonçalves M, Nosi D, Rossi Degl'Innocenti D (2017). Schwann cell TRPA1 mediates neuroinflammation that sustains macrophage-dependent neuropathic pain in mice. Nat Commun.

[8] Dobin A, Davis CA, Schlesinger F, Drenkow J, Zaleski C, Jha S (2013). STAR: ultrafast universal RNA-seq aligner. Bioinformatics.

[9] Egaña-Gorroño L, López-Díez R, Yepuri G, Ramirez LS, Reverdatto S, Gugger PF (2020). Receptor for advanced glycation end products (RAGE) and mechanisms and therapeutic opportunities in diabetes and cardiovascular disease: insights from human subjects and animal models. Front Cardiovasc Med.

[10] Figlia G, Norrmén C, Pereira JA, Gerber D, Suter U (2017). Dual function of the PI3K-Akt-mTORC1 axis in myelination of the peripheral nervous system. Elife.

[11] Gan KJ, Südhof TC (2020). SPARCL1 promotes excitatory but not inhibitory synapse formation and function independent of neurexins and neuroligins. J Neurosci.

[12] Gaudet AD, Popovich PG, Ramer MS (2011). Wallerian degeneration: gaining perspective on inflammatory events after peripheral nerve injury. J Neuroinflamm.

[13] Gerber D, Pereira JA, Gerber J, Tan G, Dimitrieva S, Yángüez E, Suter U (2021). Transcriptional profiling of mouse peripheral nerves to the single-cell level to build a sciatic nerve ATlas (SNAT). Elife.

[14] Goldberg JL, Vargas ME, Wang JT, Mandemakers W, Oster SF, Sretavan DW, Barres BA (2004). An oligodendrocyte lineage-specific semaphorin, Sema5A, inhibits axon growth by retinal ganglion cells. J Neurosci.

[15] Gomez-Sanchez JA, Carty L, Iruarrizaga-Lejarreta M, Palomo-Irigoyen M, Varela-Rey M, Griffith M (2015). Schwann cell autophagy, myelinophagy, initiates myelin clearance from injured nerves. J Cell Biol.

[16] Gomez-Sanchez JA, Pilch KS, van der Lans M, Fazal SV, Benito C, Wagstaff LJ (2017). After nerve injury, lineage tracing shows that myelin and remak Schwann cells elongate extensively and branch to form repair Schwann cells, which shorten radically on remyelination. J Neurosci.

[17] Ishii A, Furusho M, Bansal R (2021). Mek/ERK1/2-MAPK and PI3K/Akt/mTOR signaling plays both independent and cooperative roles in Schwann cell differentiation, myelination and dysmyelination. Glia.

[18] Jessen KR, Arthur-Farraj P (2019). Repair Schwann cell update: adaptive reprogramming, EMT, and stemness in regenerating nerves. Glia.

[19] Jessen KR, Mirsky R (1992). Schwann cells: early lineage, regulation of proliferation and control of myelin formation. Curr Opin Neurobiol.

[20] Jessen KR, Mirsky R (2008). Negative regulation of myelination: relevance for development, injury, and demyelinating disease. Glia.

[21] Jessen KR, Mirsky R (2016). The repair Schwann cell and its function in regenerating nerves. J Physiol.

[22] Kalinski AL, Yoon C, Huffman LD, Duncker PC, Kohen R, Passino R (2020). Analysis of the immune response to sciatic nerve injury identifies efferocytosis as a key mechanism of nerve debridement. Elife.

[23] Kucukdereli H, Allen NJ, Lee AT, Feng A, Ozlu MI, Conatser LM (2011). Control of excitatory CNS synaptogenesis by astrocyte-secreted proteins Hevin and SPARC. Proc Natl Acad Sci USA.

[24] Langfelder P, Horvath S (2012). Fast R Functions for Robust Correlations and Hierarchical Clustering. J Stat Softw..

[25] Langfelder P, Horvath S (2008). WGCNA: an R package for weighted correlation network analysis. BMC Bioinform..

[26] Li B, Dewey CN (2011). RSEM: accurate transcript quantification from RNA-Seq data with or without a reference genome. BMC Bioinform.

[27] Liu JH, Tang Q, Liu XX, Qi J, Zeng RX, Zhu ZW (2018). Analysis of transcriptome sequencing of sciatic nerves in Sprague-Dawley rats of different ages. Neural Regen Res.

[28] Love MI, Huber W, Anders S (2014). Moderated estimation of fold change and dispersion for RNA-seq data with DESeq2. Genome Biol.

[29] Lutz AB (2014). Purification of Schwann cells. Cold Spring Harb Protoc.

[30] Ma CH, Omura T, Cobos EJ, Latrémolière A, Ghasemlou N, Brenner WCJ (2011). Accelerating axonal growth promotes motor recovery after peripheral nerve injury in mice. J Clin Invest..

[31] Meyer Zu Reckendorf S, Brand C, Pedro MT, Hegler J, Schilling CS, Lerner R (2020). Lipid metabolism adaptations are reduced in human compared to murine Schwann cells following injury. Nat Commun.

[32] Nagarajan R, Le N, Mahoney H, Araki T, Milbrandt J (2002). Deciphering peripheral nerve myelination by using Schwann cell expression profiling. Proc Natl Acad Sci USA.

[33] Napoli I, Noon LA, Ribeiro S, Kerai AP, Parrinello S, Rosenberg LH (2012). A central role for the ERK-signaling pathway in controlling Schwann cell plasticity and peripheral nerve regeneration in vivo. Neuron.

[34] Narciso MS, Mietto Bde S, Marques SA, Soares CP, Mermelstein Cdos S, El-Cheikh MC, Martinez AM (2009). Sciatic nerve regeneration is accelerated in galectin-3 knockout mice. Exp Neurol.

[35] Newbern JM, Li X, Shoemaker SE, Zhou J, Zhong J, Wu Y (2011). Specific functions for ERK/MAPK signaling during PNS development. Neuron.

[36] Painter MW, Brosius Lutz A, Cheng YC, Latremoliere A, Duong K, Miller CM (2014). Diminished Schwann cell repair responses underlie age-associated impaired axonal regeneration. Neuron.

[37] Reichert F, Saada A, Rotshenker S (1994). Peripheral nerve injury induces Schwann cells to express two macrophage phenotypes: phagocytosis and the galactose-specific lectin MAC-2. J Neurosci.

[38] Senatus LM, Schmidt AM (2017). The AGE–RAGE axis: implications for age-associated arterial diseases. Front Genet.

[39] Tasdemir-Yilmaz OE, Druckenbrod NR, Olukoya OO, Dong W, Yung AR, Bastille I, Segal RA (2021). Diversity of developing peripheral glia revealed by single-cell RNA sequencing. Dev Cell.

[40] Toma JS, Karamboulas K, Carr MJ, Kolaj A, Yuzwa SA, Mahmud N (2020). Peripheral nerve single-cell analysis identifies mesenchymal ligands that promote axonal growth. eNeuro.

[41] Ulgen E, Ozisik O, Sezerman OU (2019). pathfindR: an R package for comprehensive identification of enriched pathways in omics data through active subnetworks. Front Genet.

[42] Wada R, Yagihashi S (2005). Role of advanced glycation end products and their receptors in development of diabetic neuropathy. Ann N Y Acad Sci.

[43] Weiss T, Taschner-Mandl S, Bileck A, Slany A, Kromp F, Rifatbegovic F, Frech C, Windhager R (2016). Proteomics and transcriptomics of peripheral nerve tissue and cells unravel new aspects of the human Schwann cell repair phenotype. Glia.

[44] Welleford AS, Quintero JE, Seblani NE, Blalock E, Gunewardena S, Shapiro SM (2020). RNA sequencing of human peripheral nerve in response to injury: distinctive analysis of the nerve repair pathways. Cell Transplant.

[45] Wilson GF, Chiu SY (1990). Ion channels in axon and Schwann cell membranes at paranodes of mammalian myelinated fibers studied with patch clamp. J Neurosci.

[46] Wolbert J, Li X, Heming M, Mausberg AK, Akkermann D, Frydrychowicz C (2020). Redefining the heterogeneity of peripheral nerve cells in health and autoimmunity. Proc Natl Acad Sci USA.

[47] Wu W, Liu Y, Wang Y (2016). Sam68 promotes Schwann cell proliferation by enhancing the PI3K/Akt pathway and acts on regeneration after sciatic nerve crush. Biochem Biophys Res Commun.

[48] Xu X, Gao W, Li L, Hao J, Yang B, Wang T (2021). Annexin A1 protects against cerebral ischemia-reperfusion injury by modulating microglia/macrophage polarization via FPR2/ALX-dependent AMPK-mTOR pathway. J Neuroinflamm.

[49] Yi S, Zhang H, Gong L, Wu J, Zha G, Zhou S (2015). Deep sequencing and bioinformatic analysis of lesioned sciatic nerves after crush injury. PLoS ONE.

[50] Zhang Y, Sloan SA, Clarke LE, Caneda C, Plaza CA, Blumenthal PD (2016). Purification and characterization of progenitor and mature human astrocytes reveals transcriptional and functional differences with mouse. Neuron..

