# Supplementary figures and images for: An RNA-sequencing transcriptome of the rodent Schwann cell response to peripheral nerve injury
Source: J Neuroinflammation. 2022 Apr 30;19:105. doi: 10.1186/s12974-022-02462-6 (PMC9063194; doi:10.1186/s12974-022-02462-6)

**A**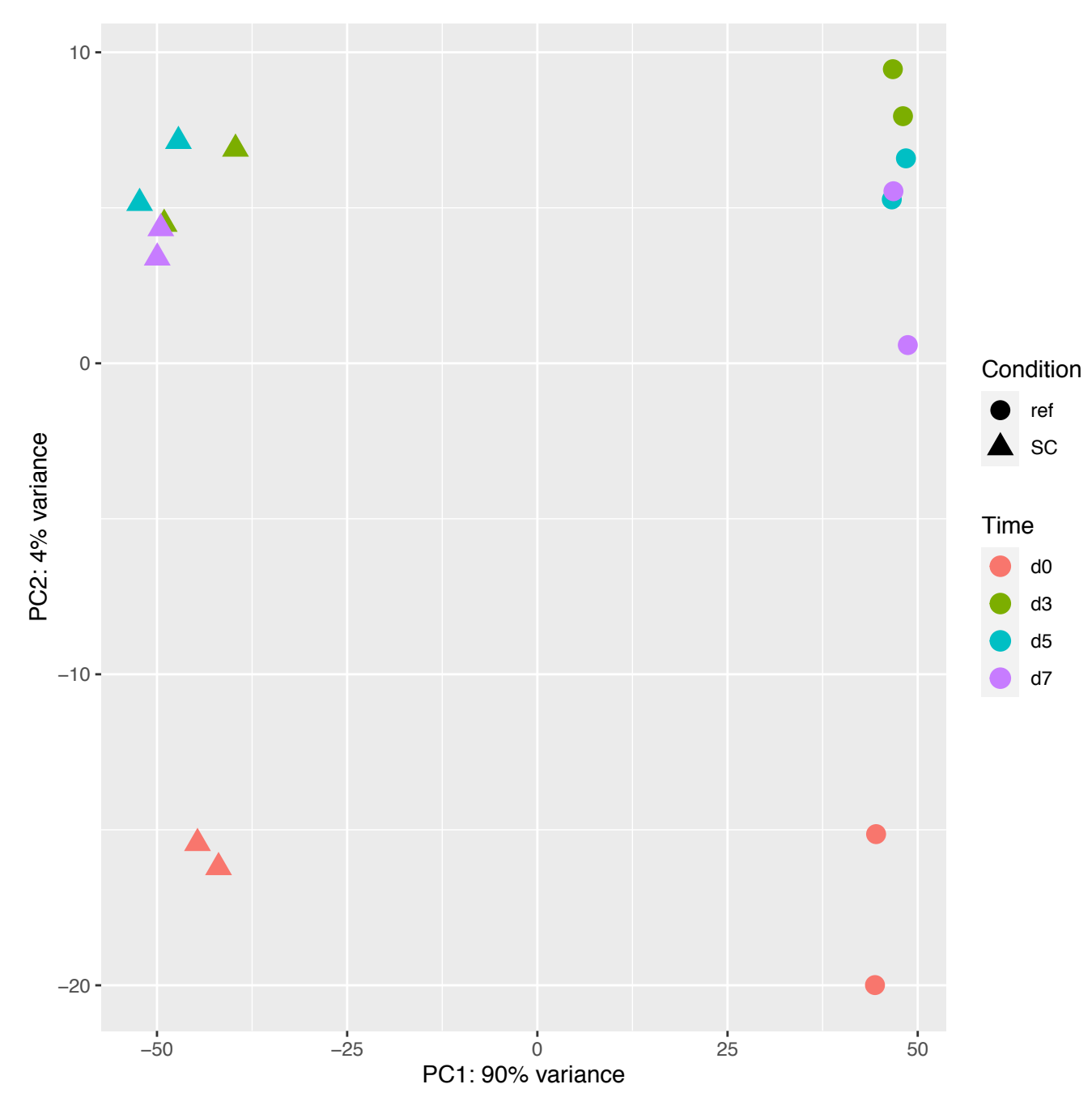**B**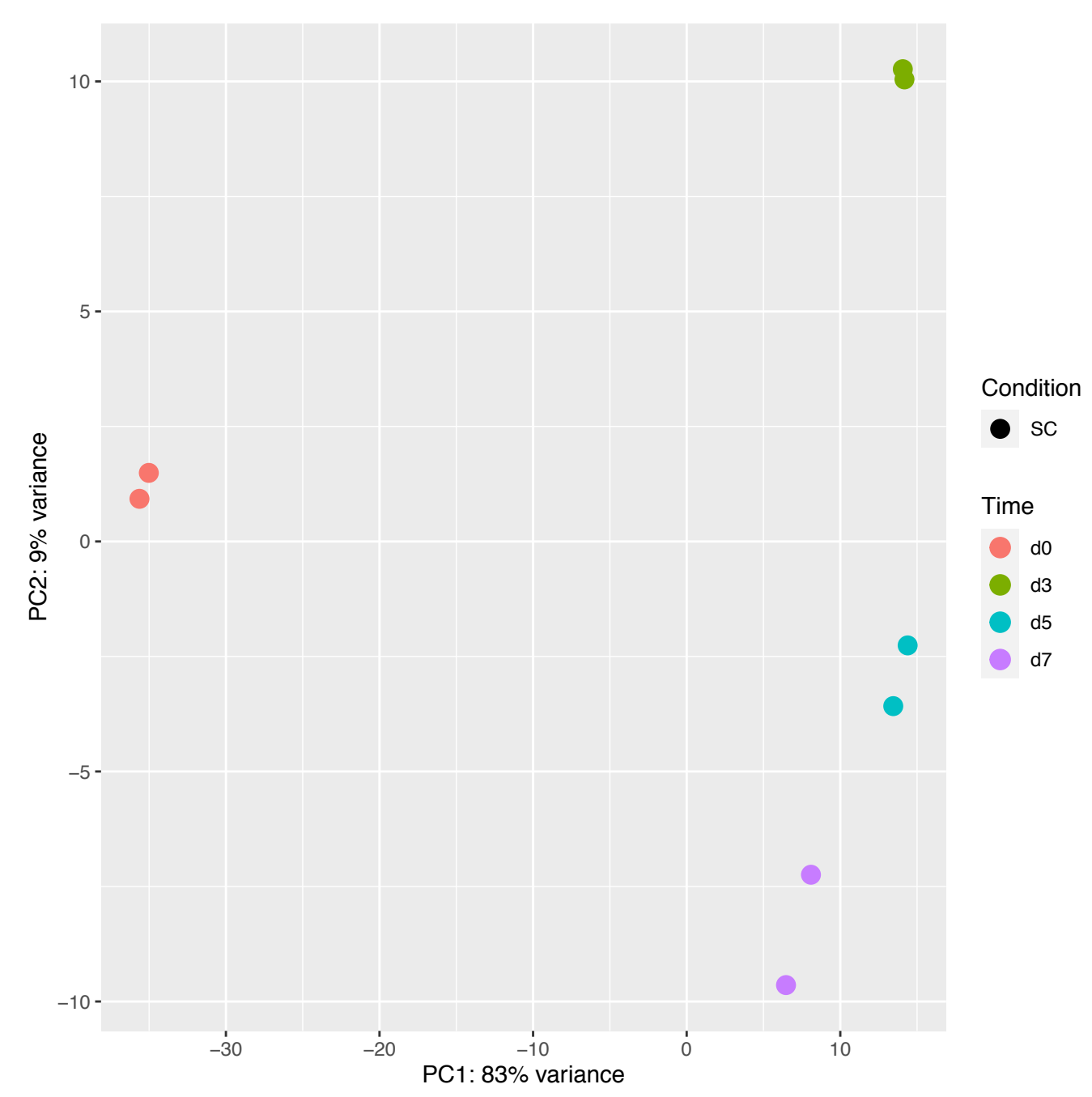

Supplement: Supplementary file 1 — Additional file 1. PCA of all RNA-seq samples. (A) PCA of all samples colored by day post-crush. Circles indicate whole nerve samples and triangles represent Schwann cell purified samples. (B) PCA of Schwann cell samples alone, colored by time point. [file 12974_2022_2462_MOESM1_ESM.pdf]

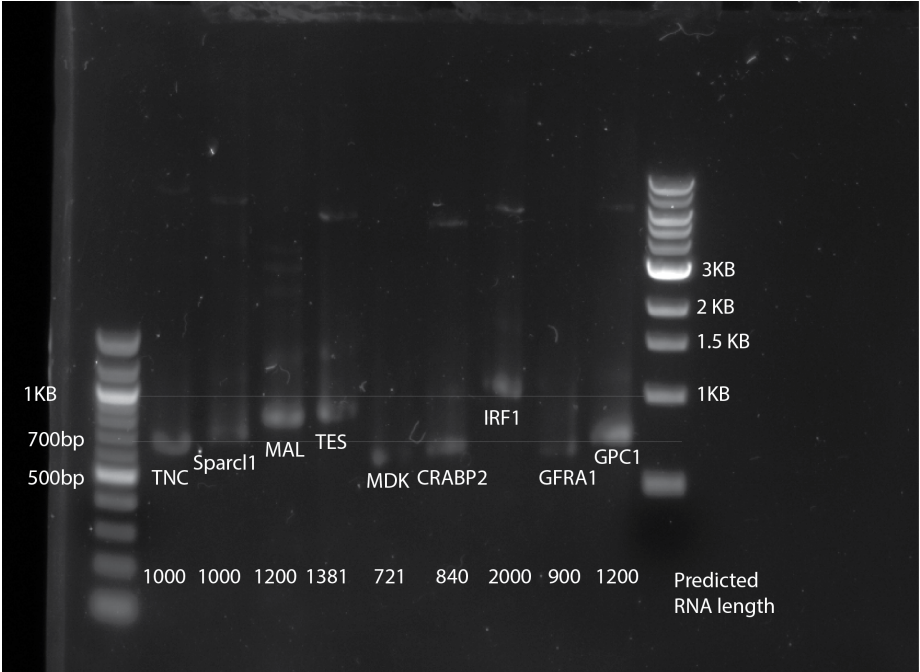

Supplement: Supplementary file 2 — Additional file 2. Generation of in situ hybridization probes. RNA probes used for in situ hybridization validation. Expected probe lengths are listed beneath each column. [file 12974_2022_2462_MOESM2_ESM.pdf]

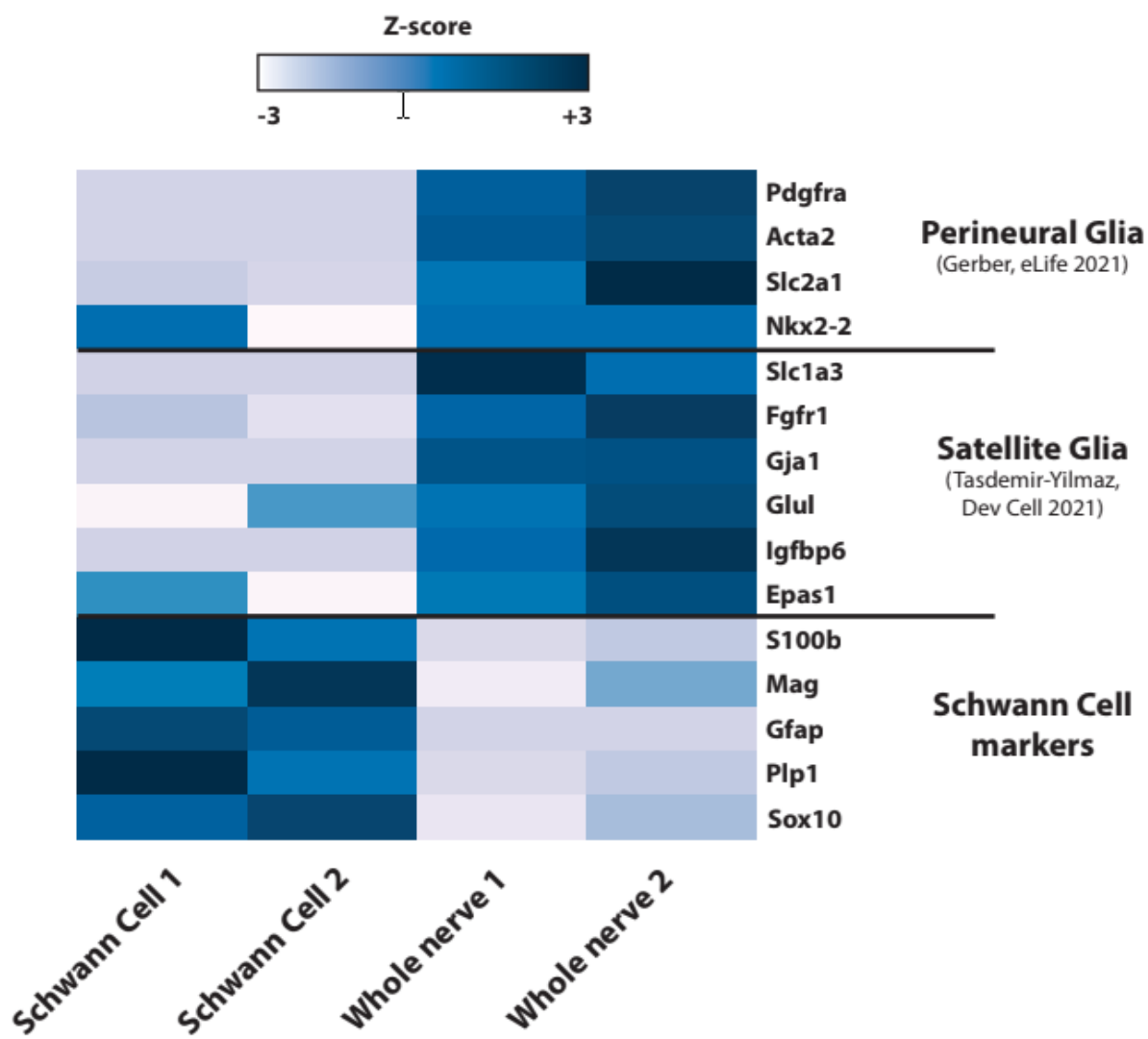

Supplement: Supplementary file 3 — Additional file 3. qRT-PCR primer sequences (rat) used to validate RNAseq findings. [file 12974_2022_2462_MOESM3_ESM.pdf]
